# Gallstone Formation Follows a Different Trajectory in Bariatric Patients Compared to Nonbariatric Patients

**DOI:** 10.3390/metabo11100682

**Published:** 2021-10-05

**Authors:** Sylke Haal, Maimoena S. S. Guman, Yair I. Z. Acherman, Johannes P. G. Jansen, Michel van Weeghel, Henk van Lenthe, Eric J. M. Wever, Victor E. A. Gerdes, Rogier P. Voermans, Albert K. Groen

**Affiliations:** 1 Department of Internal Medicine, Spaane Gasthuis, 2134 TM Hoofddorp, The Netherlands; m.s.guman@amsterdamumc.nl (M.S.S.G.); v.e.gerdes@amsterdamumc.nl (V.E.A.G.); 2 Department of Gastroenterology and Hepatology, Amsterdam UMC, University of Amsterdam, Amsterdam Gastroenterology Endocrinology Metabolism, 1105 AZ Amsterdam, The Netherlands; r.p.voermans@amsterdamumc.nl; 3 Department of Internal and Vascular Medicine, Amsterdam UMC, University of Amsterdam, Amsterdam Gastroenterology Endocrinology Metabolism, 1105 AZ Amsterdam, The Netherlands; j.p.jansen@amsterdamumc.nl (J.P.G.J.); a.k.groen@amsterdamumc.nl (A.K.G.); 4 Department of Surgery, Spaarne Gasthuis, 2134 TM Hoofddorp, The Netherlands; yacherman@spaarnegasthuis.nl; 5 Laboratory of Genetic Metabolic Diseases, Amsterdam UMC, University of Amsterdam, Amsterdam Gastroenterology Endocrinology Metabolism, Amsterdam Cardiovascular Sciences, 1105 AZ Amsterdam, The Netherlands; m.vanweeghel@amsterdamumc.nl (M.v.W.); h.vanlenthe@amsterdamumc.nl (H.v.L.); eric.wever@amsterdamumc.nl (E.J.M.W.); 6 Core Facility Metabolomics, Amsterdam UMC, University of Amsterdam, 1105 AZ Amsterdam, The Netherlands; 7 Bioinformatics Laboratory, Department of Epidemiology and Data Science, Amsterdam UMC, University of Amsterdam, Amsterdam Public Health, 1105 AZ Amsterdam, The Netherlands

**Keywords:** gallstones, bariatric surgery, bile, triglyceride-rich lipoproteins, lipidomics

## Abstract

Since obese patients form cholesterol gallstones very rapidly after bariatric surgery, in patients who did not form gallstones during preceding years, we hypothesized that gallstone formation follows a different trajectory in bariatric patients compared to nonbariatric patients. We therefore analyzed the lipid composition of gallbladder bile derived from 18 bariatric gallstone patients and 17 nonbariatric gallstone patients (median (IQR) age, 46.0 (28.0–54.0) years; 33 (94%) female) during laparoscopic cholecystectomy using an enzymatic and lipidomics approach. We observed a higher concentration of total lipids (9.9 vs. 5.8 g/dL), bile acids (157.7 vs. 81.5 mM), cholesterol (10.6 vs. 5.4 mM), and phospholipids (30.4 vs. 21.8 mM) in bariatric gallstone patients compared to nonbariatric gallstone patients. The cholesterol saturation index did not significantly differ between the two groups. Lipidomics analysis revealed an interesting pattern. Enhanced amounts of a number of lipid species were found in the gallbladder bile of nonbariatric gallstone patients. Most striking was a fivefold higher amount of triglyceride. A concomitant ninefold increase of apolipoprotein B was found, suggesting secretion of triglyceride-rich lipoproteins (TRLs) at the canalicular pole of the hepatocyte in livers from nonbariatric gallstone patients. These findings suggest that gallstone formation follows a different trajectory in bariatric patients compared to nonbariatric patients. Impaired gallbladder emptying might explain the rapid gallstone formation after bariatric surgery, while biliary TRL secretion might contribute to gallstone formation in nonbariatric patients.

## 1. Introduction

Cholesterol gallstone disease is a prevalent and multifactorial disease. The formation of cholesterol gallstones implies a failure of biliary cholesterol homeostasis [1]. Involved factors include unbalanced hepatic biliary lipid secretion leading to bile that is supersaturated with cholesterol, the presence of crystallization-promoting factors, impaired gallbladder contraction, altered intestinal lipid and bile acid absorption, and altered gut microbiota. In addition, multiple lithogenic gene variations are identified that are involved in the regulation of cholesterol homeostasis [2]. Although supersaturated bile is a prerequisite for the formation of cholesterol gallstones, it is also frequently found in patients without gallstones [3,4]. Accordingly, the nucleation of cholesterol crystals from supersaturated bile is considered to be the critical step in the formation of cholesterol gallstones.

Obese patients undergoing bariatric surgery are at high risk for the formation of cholesterol gallstones. Up to 40% of bariatric patients develop gallstones [5,6,7], and approximately 8% to 15% become symptomatic [8,9,10]. Most gallstones are formed within 6 months after bariatric surgery [11], and cholecystectomy is often performed within 2 years [12,13]. Since obese patients form cholesterol gallstones very rapidly after bariatric surgery, in patients who did not form gallstones during preceding years, we hypothesized that gallstone formation follows a different trajectory in bariatric patients compared to nonbariatric patients.

Several studies have investigated the biliary lipid composition in obese patients undergoing (mostly diet-induced) weight loss using duodenal bile samples [14,15,16,17,18,19,20,21,22,23,24,25]. Most studies showed that the cholesterol saturation index (CSI) of bile further increases during weight loss, although the results varied with the duration of follow-up [14,15,16,17,18,19,20]. In two studies, no significant changes in CSI were observed [21,24]. Nevertheless, these studies were not able to demonstrate whether their observed alterations preceded gallstone formation, because the number of patients who formed gallstones were scarce. Two other studies investigated changes in biliary lipid composition following gallstone formation after bariatric surgery [22,23,25]. Both studies observed that the bile acid concentration was increased (only significantly in one study) in patients who formed gallstones after weight reduction, while the concentration of cholesterol, phospholipids and the CSI were not significantly altered.

In the present study, we analyzed the lipid composition of gallbladder bile derived from bariatric and nonbariatric gallstone patients during laparoscopic cholecystectomy. For the first time, we carried out a full lipidomics analysis of bile in these patient groups. We observed significant differences in the common biliary lipids as well as minor lipid species.

## 2. Results

### 2.1. Study Population

The study population consisted of 35 patients with symptomatic gallstones, of which 18 patients had undergone laparoscopic Roux-en-Y gastric bypass previously. Their characteristics are summarized in Table 1. Bariatric gallstone patients were younger than nonbariatric gallstone patients. In both groups, most patients were female and the body mass index on the day of cholecystectomy was comparable. None of the bariatric gallstone patients used lipid-lowering drugs at the time of cholecystectomy, while two nonbariatric gallstone patients used a statin.

### 2.2. Stones

All bariatric patients had multiple small cholesterol gallstones varying in size from 1 to 3 mm. Most nonbariatric patients had large cholesterol stones varying in number and size from 10 to 40 mm.

### 2.3. Biliary Lipid Composition, Cholesterol Saturation, and Cholesterol Crystals

**Figure 1:**
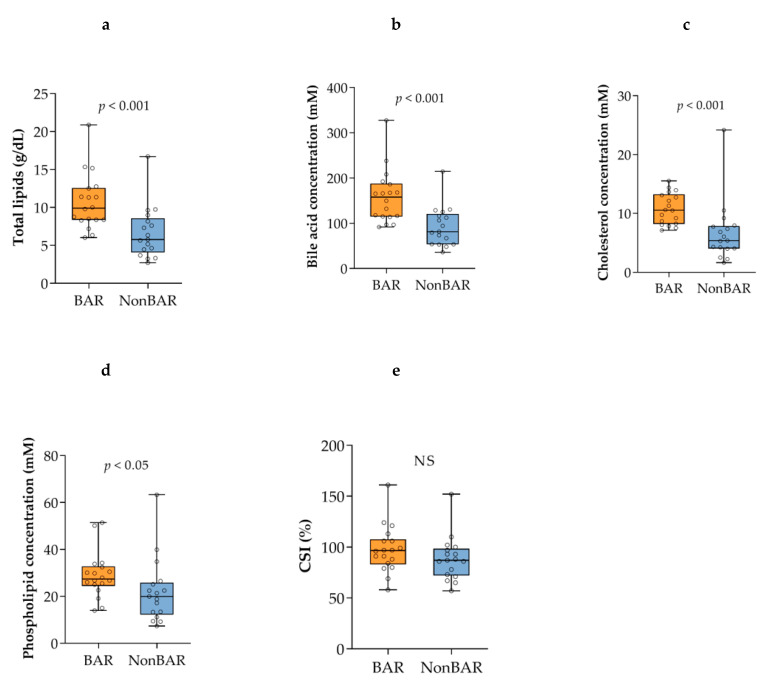
Lipids in gallbladder bile are shown as box and whisker plots, with orange plots indicating bariatric gallstone patients, and blue plots indicating gallstone patients: (**a**) total lipids; (**b**) bile acid; (**c**) cholesterol; (**d**) phospholipid; (**e**) CSI. The horizontal line indicates the median, box borders indicate the lower and upper quartiles, whiskers indicate the minimum and maximum values, and the dots indicate the individual values. CSI: cholesterol saturation index, NS: non-significant.

The total lipid concentration (9.9 vs. 5.8 g/dL; *p* < 0.001) was higher in bariatric gallstone patients compared to nonbariatric gallstone patients (Figure 1). Concordantly, a much higher median bile acid concentration (157.7 vs. 81.5 millimolar (mM); *p* < 0.001), higher cholesterol concentration (10.6 vs. 5.4 mM; *p* < 0.001), and higher phospholipid concentration (30.4 vs. 21.8 mM; *p* < 0.05) were found in bariatric gallstone patients. The CSI did not differ significantly between the groups (96.5 vs. 87.0%; *p* = 0.15). In both groups, most gallbladder biles contained cholesterol crystals (15/18 vs. 15/17).

### 2.4. Lipidomics

**Figure 2:**
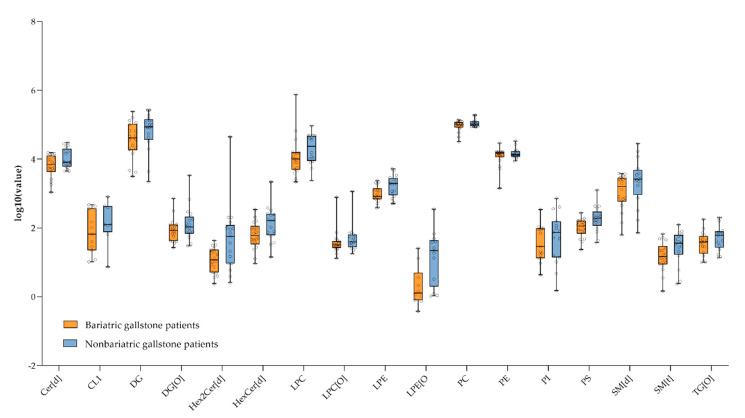
Logarithm-transformed total lipid levels in gallbladder bile sorted by lipid class that did not significantly differ between the groups. Total levels are defined as the ratio between the abundance of all lipid species of the same class and the abundance of the corresponding internal standard. Cer[d], ceramide; CL1, cardiolipin; DG, diglyceride; DG[O], alkyl-diglyceride; Hex2Cer[d], dihexosylceramide; HexCer[d], hexosylceramide; LPC, lyso-phosphatidylcholine; LPC[O], (lyso)alkyl-phosphatidylcholine; LPE, lyso-phosphatidylethanolamine; LPE[O], (lyso)alkyl-phosphatidylethanolamine; PC, phosphatidylcholine; PE, phosphatidylethanolamine; PI, phosphatidylinositol; PS, phosphatidylserine; SM[d], sphingomyelin; SM[t], hydroxysphingomyelin; and TG[O], alkyl-triglyceride.

**Figure 3:**
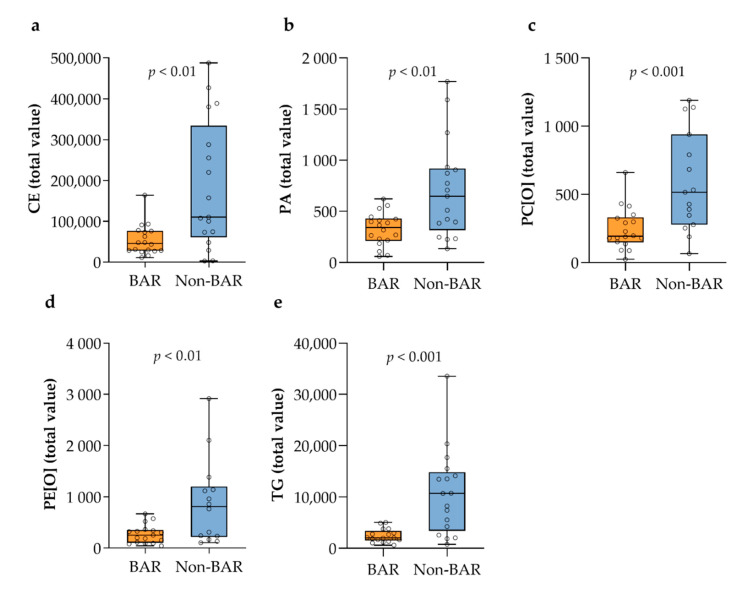
Total lipid levels in gallbladder bile sorted by lipid class that differed significantly between the groups: (**a**) cholesterylester; (**b**) phosphatidic acid; (**c**) alkyl-phosphatidylcholine; (**d**) alkyl-phosphatidylethanolamine; (**e**) triglyceride.

**Figure 4:**
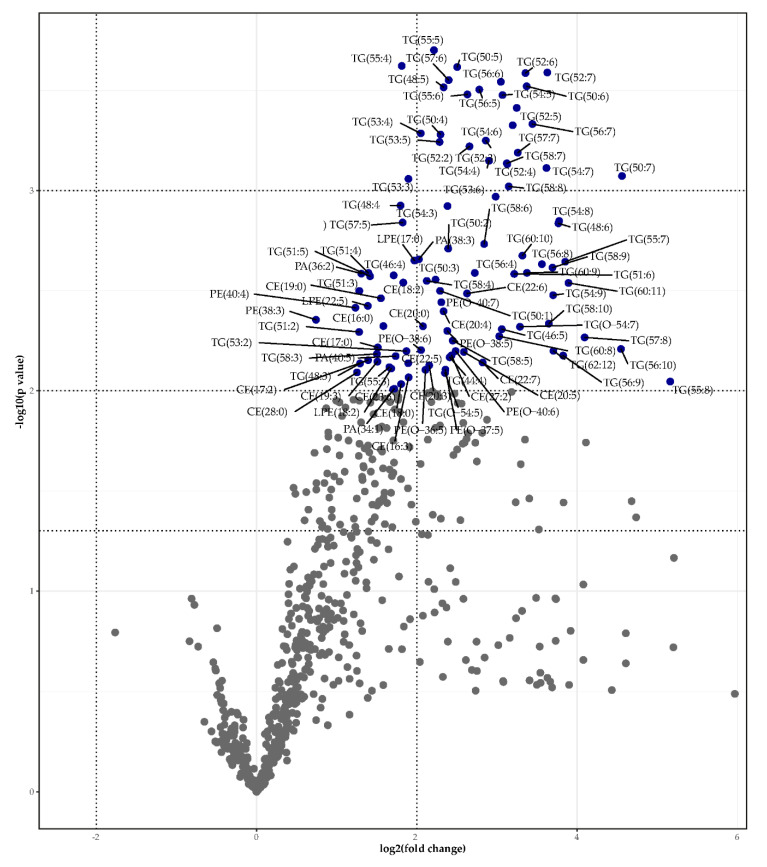
Volcano plot (significance (-log10(*p*-value)) versus effect size (log2(fold change)) depicting the lipidomics data. The most discriminative lipid species based on *p*-value are labeled. The three horizontal lines indicate *p*-values of 0.05, 0.01, and 0.001, respectively. The two vertical dotted lines indicate log2(fold change) of −2 and 2, respectively.

**Figure 5:**
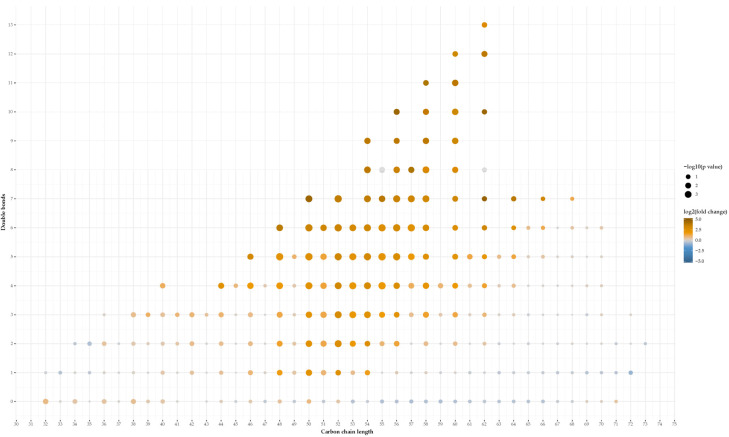
Plot (carbon chain length versus double bounds) of the triglyceride species found in gallbladder bile from bariatric gallstone patients compared to nonbariatric gallstone patients. Significance (-log10(*p*-value)) and effect size (log2(fold change)) are shown in the legend.

We identified four lipid categories (sterols, sphingolipids, glycerolipids, and glycerophospholipids), 22 main lipid classes, and within these classes, 1000 different lipid species (Supplemental Table S1). The partial least squares regression discriminant analysis is shown in Supplemental Figure S1. The total lipid levels of most lipid classes did not differ significantly between the two groups (Figure 2). The total levels of cholesterylester (CE), phosphatidic acid (PA), alkyl-phosphatidylcholine (PC[O]), alkyl-phosphatidylethanolamine (PE[O]), and especially triglyceride (TG) were significantly lower in bariatric gallstone patients compared to nonbariatric gallstone patients (Figure 3). The most discriminative species included mainly TG species (Figure 4 and Supplemental Figure S2). Figure 4 and Figure 5 show a striking presence of TG species constituted of polyunsaturated long-chain species in nonbariatric gallstone patients.

### 2.5. Apolipoproteins

**Figure 6:**
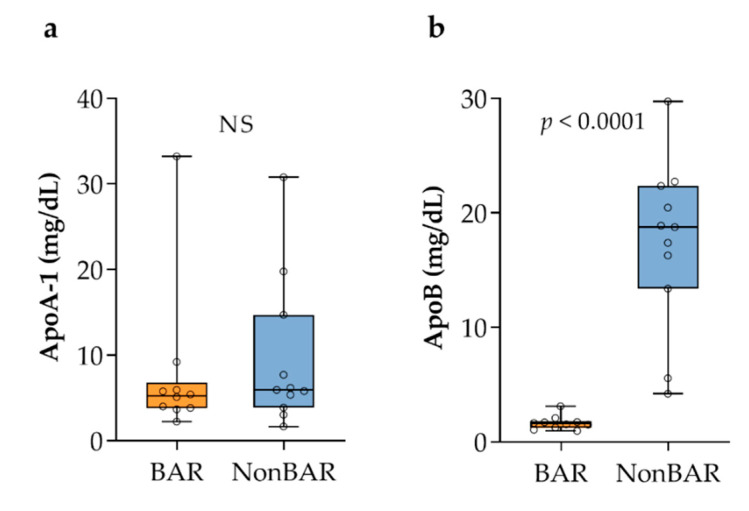
Apolipoproteins in gallbladder bile: (**a**) ApoA-1; (**b**) ApoB.

Finding enhanced amounts of CE and TG in gallbladder biles from nonbariatric gallstone patients was unexpected. Since CE and TG are solubilized in plasma in the form of lipoproteins, we investigated whether concentrations of apolipoprotein (apo) A-1 and apoB in the gallbladder bile of the two groups associated with the difference in CE and TG. The concentration of apoA-1 did not significantly differ (5.3 vs. 5.9 mg/dL; *p* = 0.47), while a ninefold lower median concentration of apoB was found in bariatric gallstone patients compared to nonbariatric gallstone patients (1.6 vs. 18.8 mg/dL; *p* < 0.0001; Figure 6).

## 3. Discussion

### 3.1. Main Findings

In the present study, we show that the biliary lipid composition of bariatric gallstone patients differs from nonbariatric gallstone patients. We observed a higher concentration of bile acids, phospholipids, and cholesterol in the gallbladder bile of bariatric gallstone patients compared to nonbariatric gallstone patients. Concordantly, a higher total lipid concentration was observed in bariatric patients. Interestingly, pronounced differences were observed in minor lipid species identified by lipidomics analysis. In particular, a fivefold higher amount of TG in nonbariatric gallstone patients was found.

### 3.2. Interpretations

Rudling et al. recently stated that supersaturation of the gallbladder bile of nonbariatric gallstone patients is mainly due to a reduced bile acid concentration instead of an increased cholesterol concentration [26]. Even though our study lacked gallstone-free bariatric patients, the much higher bile acid concentration in bariatric gallstone patients refutes the hypothesis that bile acid deficiency plays a major role in bile supersaturation after bariatric surgery and subsequent gallstone formation. In both patient groups, the observed median CSIs indicate that most gallbladder biles were not supersaturated at the time of cholecystectomy. However, since bile supersaturation is a prerequisite for gallstone formation, we assume that it must have been present in an earlier phase. In obesity, supersaturation is mainly attributed to an increased cholesterol secretion [14,27], but during weight loss, the secretion of all three biliary lipids appears to be reduced [14,24]. A common explanation for supersaturation during weight loss has been the mobilization of cholesterol from adipose tissue stores [14,17,18,23,24]. Although still often mentioned, this hypothesis seems unlikely since lipid droplets contain little cholesterol. Hence, lipid mobilization almost exclusively targets TGs that are released as fatty acids during fasting [28]. Our finding of high concentrations of all three biliary lipids at the time of cholecystectomy following gastric bypass surgery is in line with the observations of Shiffman et al. [22,23,25]. Similarly, Gustaffson et al. found an increased total biliary lipid concentration in 25 obese patients after vertical banded gastroplasty, regardless of gallstone formation [18].

Based on our finding of a high total lipid concentration and a large number of small stones in bariatric gallstone patients, we speculate that gallstone formation after bariatric surgery is primarily due to impaired gallbladder emptying which provides the time and condition for the nucleation of cholesterol crystals. First, a prolonged residence time results in more concentrated gallbladder bile, which is known to facilitate cholesterol crystallization [29,30,31,32]. Second, in the case of impaired gallbladder emptying, crystals have the time to aggregate and grow into stones, whereas in case of adequate gallbladder emptying, they are released into the duodenum. Bastouly et al. demonstrated that postprandial gallbladder emptying was significantly compromised after Roux-en-Y gastric bypass, as demonstrated by an increased residual volume and decreased maximum ejection fraction [33]. Similarly, studies have shown that gallbladder emptying is decreased in obese patients on low-fat low-calorie diets [16,17]. Furthermore, Stolk and colleagues have studied gallbladder emptying, gallbladder bile composition and cholesterol nucleation in nonbariatric gallstone patients [34]. They have shown that weak gallbladder contractors (patients with less than 50% of maximal postprandial emptying) had much higher bile acid and phospholipid concentrations compared to strong contractors, while cholesterol concentrations were comparable. Consequently, total lipid concentration was higher and CSI was lower. Taking this evidence together, the trajectory via which bariatric patients develop gallstones seems straightforward and can be prevented by drugs such as ursodeoxycholic acid [35]. The question of how nonbariatric patients develop gallstones at relatively low bile salt and, hence, low total lipid concentration remains unanswered. However, we feel that our full lipidomics analysis provides new insights. A lipidomics analysis of bile was carried out in previous studies attempting to differentiate between PSC patients and controls [36] or cancer patients and controls [37]. Many differences between the diseased patients and controls were found in these studies. In contrast, in our study, the differences between the two gallstone patient groups focused on a few lipid classes. Particularly, the differences in CE and TG were striking, and the question arises as to where these lipids originated. The most obvious sources are the liver and the plasma compartment. In the liver, CE and TG are stored in lipid droplets, and in plasma, CE and TG are found in high concentrations in triglyceride-rich lipoproteins (TRLs). The association between apoB vs. CE and TG suggests that TRLs are the source. Whether they originate in the liver and entered the bile via misrouting or came to the bile via transcytosis remains to be investigated. We hypothesize that PA, PC[O], PE[O], and the other lipids showing a higher presence in bile of nonbariatric gallstone patients arrived piggybacked on TRLs. The relatively high concentration of apoB in bile was reported about 40 years ago [38], but whether it plays a functional role in biliary cholesterol solubilization has not been investigated yet.

### 3.3. Limitations

Our findings should be interpreted by taking into account the small sample size, lack of control groups of gallstone-free patients, and single sampling (only after gallstone formation). As a result, it remains to be determined if the observed differences in lipid composition are unique to bariatric gallstone patients or occur in all bariatric patients. Furthermore, the fact that the composition of gallbladder bile is not static but continuously changing should also be taken into consideration. Hence, these limitations do not allow us to draw firm conclusions on cause–effect relationships.

### 3.4. Conclusion

The present study has shown that: (1) the lipid composition of gallbladder bile differs between bariatric gallstone patients and nonbariatric gallstone patients, (2) this finding suggests that gallstone formation follows a different trajectory in bariatric patients compared to nonbariatric patients, and (3) impaired gallbladder emptying might explain the rapid gallstone formation after bariatric surgery, whereas biliary TRL secretion might contribute to gallstone formation in nonbariatric patients.

## 4. Materials and Methods

### 4.1. Patients

Patients aged 18 years and over with symptomatic gallstone disease scheduled for elective laparoscopic cholecystectomy at the Spaarne Gasthuis (Hoofddorp, The Netherlands) were included. Patients with a Roux-en-Y gastric bypass (bariatric patients) were eligible for inclusion if the cholecystectomy was performed within four years after bariatric surgery. Only gallbladder biles from cholesterol gallstone patients with a total biliary lipid concentration of at least 3 grams per deciliter were further analyzed in this study. Exclusion criteria were inability or refusal to give informed consent, suspicion of malignancy and active/acute gallbladder inflammation (cholecystitis). An additional exclusion criterion for nonbariatric patients was a substantial amount of weight loss prior to cholecystectomy. The ethical committee of the Slotervaart Hospital and Reade (Amsterdam, The Netherlands) confirmed that the Dutch Medical Research Involving Human Subjects Act did not apply to the current study and that an official approval was not required. This study was conducted in accordance with the Declaration of Helsinki after the study protocol was approved by the board of directors of the Spaarne Gasthuis. All patients provided written informed consent.

### 4.2. Sample Collection

After cholecystectomy, gallbladder bile samples were collected in 2 mL tubes and all but one were immediately frozen at −80 °C. Gallstones were extracted from the gallbladder, washed, and preserved in dried condition.

### 4.3. Gallstone Composition

Gallstones were classified as cholesterol stones by visual inspection and confirmed by biochemical analysis. Finely powdered stone samples (range of 14–99 mg) were dissolved in 1.0 mL isopropanol 99.99%. Sample cholesterol concentrations were determined using a cholesterol kit for Selectra from DiaSys Diagnostic Systems (Holzheim, Germany). Stones containing 70% or more cholesterol were defined as cholesterol stones [39].

### 4.4. Biliary Lipid Composition, CSI, Cholesterol Crystals, and Apolipoproteins

After thawing and centrifugation of the gallbladder bile sample, 10 µL of bile was extracted and diluted (1:300 and 1:600). Concentrations of free cholesterol, phospholipids and bile acids were determined using a fluorometric enzymatic method [40]. All measurements were conducted on a CLARIOstar analyzer (BMG Labtech, Ortenberg, Germany). Total lipid concentration and CSI were calculated based on the critical tables by Carey [41]. Fresh gallbladder bile samples were examined for typical rhomboid monohydrate cholesterol crystals by light microscopy. Apolipoprotein A-1 and apolipoprotein B were measured with immunoturbidimetric tests from DiaSys Diagnostic Systems (Holzheim, Germany).

### 4.5. Lipidomics Analysis

#### 4.5.1. One-Phase Lipidomic Extraction

In a 2 mL tube, bile and internal standards corrected for total phospholipid concentration were added. After addition of the bile and internal standard, 1.5 mL 1:1 (*v*/*v*) methanol/chloroform was added. Samples were thoroughly mixed for 5 min and centrifuged for 5 min at 20,000× *g*. The supernatant was transferred to a 4 mL glass vial and evaporated under a stream of nitrogen at 45 °C, the residue was dissolved in an adjusted amount of 1:1 (*v*/*v*) methanol/chloroform, mixed thoroughly, and 150 µL transferred to a glass HPLC vial.

#### 4.5.2. Internal Standards

A set of internal standards (purchased from Avanti Polar Lipids, Alabaster, AL, USA) was added to each sample after sample workup and before data collection. The concentrations of internal standards are in mmol/mol biliary phospholipids (i.e., samples with a high phospholipid concentration received a high concentration of internal standard, and vice versa): diglyceride (14:0)_2_ (1 mmol/mol), triglyceride (TG) (14:0)_3_ (1 mmol/mol), cholesterylester (CE) (16:0)-d7 (5 mmol/mol), cardiolipin (14:0)_4_ (0.2 mmol/mol), bis(monoacylglycero)phosphate (14:0)_2_ (0.4 mmol/mol), phosphatidylcholine (14:0)_2_ (4 mmol/mol), phosphatidylglycerol (14:0)_2_ (0.2 mmol/mol), phosphatidylserine (14:0)_2_ (10 mmol/mol), phosphatidylethanolamine (14:0)_2_ (1 mmol/mol), phosphatidic acid (PA) (14:0)_2_ (1 mmol/mol), phosphatidylinositol (8:0)_2_ (1 mmol/mol), sphingomyelin (12:0) (4.25 mmol/mol), lyso-phosphatidylglycerol (14:0) (0.2 mmol/mol), lyso-phosphatidylethanolamine (14:0) (1 mmol/mol), lyso-phosphatidylcholine (14:0) (2 mmol/mol), lyso-phosphatidic acid (14:0) (0.2 mmol/mol), sphingosine (d17:0 and 17:1) (0.25 mmol/mol), sphingosine 1-phophate (d17:0 and 17:1) (0.25 mmol/mol), lactosylceramide (d18:1/12:0) (0.25 mmol/mol), glucosylceramide (d18:1/12:0) (0.25 mmol/mol), ceramide (d18:1/12:0 and d18:1/25:0) (0.25 mmol/mol), and ceramide 1-phophate (d18:1/12:0) (0.25 mmol/mol).

#### 4.5.3. Method

Lipidomics analysis was performed as described previously with the most important steps and minor changes described in this section [42]. The HPLC system consisted of an UltiMate 3000 binary HPLC pump, a vacuum degasser, a column temperature controller, and an auto sampler (Thermo Scientific, Waltham, MA, USA). Lipid extract (5 µL) was injected onto a “normal phase column” Phenomenex LUNA silica 2 × 250 mm, 5 µm particle diameter (Merck, Darmstadt, Germany) and a “reverse phase column” Acquity UPLC HSS T3, 1.8 µm particle diameter (Waters, Milford, MA, USA). A Q Exactive Plus Orbitrap (Thermo Scientific) mass spectrometer was used in the negative and positive electrospray ionization mode. In both ionization modes, mass spectra of the lipid species were obtained by continuous scanning from m/z 200 to m/z 2000 with a resolution of 280,000 full width at half maximum. Nitrogen was used as nebulizing gas. The spray voltage used was 2500 V, the capillary temperature was 256 °C, S-lens RF level was 50, auxiliary gas level was 11, auxiliary gas temperature was 300 °C, sheath gas level was 48, and sweep cone gas level was 2.

#### 4.5.4. Processing

An in-house developed pipeline, written in the R programming language, was used for data processing. The raw data files were converted to mzXML using MSconvert in centroided mode [43]. Peak finding and peak group finding was conducted using the R package XCMS, with minor modifications to some functions for a better representation of the Q Exactive data. Annotation of the peaks was conducted based on an in-house database containing all possible lipid species.

#### 4.5.5. Identification

Identification of metabolites was based on exact mass (with 3 ppm tolerance) and retention time, including the relation between these two parameters, taking into account the different molecular species of the lipid class.

### 4.6. Statistical Analysis

Descriptive statistics were used to describe the study population. Continuous variables with a skewed distribution were summarized using medians and interquartile ranges. The Mann–Whitney U test was used to compare the biliary lipid composition between the two groups. We considered a two-sided *p*-value of less than 0.05 to be statistically significant. Statistical analyses were performed using SPSS statistics for Windows (version 26, Armonk, NY, USA: IBMP Corp.).

#### Lipidomics Data

Partial least squares regression discriminant analysis was performed to examine whether significant differences existed among the two groups. The total levels of the lipid species (the summations of the different species of the same main class) are defined as the ratio between the abundance of all lipid species of the same class and the abundance of the corresponding internal standard and presented in box and whisker plots. Data in the box and whisker plots are presented as a median and interquartile range, together with the maximum, minimum, and individual values. The Mann–Whitney U test was used for statistical comparison of the total lipid species between the two groups. We considered a two-sided *p*-value of less than 0.01 to be statistically significant.

## Figures and Tables

**Table 1:** Patient characteristics.

	ALL (*n* = 35)	BAR ^1^ (*n* = 18)	NonBAR^2^ (*n* = 17)
Age in years	46.0 [28.0–54.0]	41.0 [25.0–47.3]	48.0 [35.5–61.0]
Female gender, *n* (%)	33 (94)	17 (94)	16 (94)
BMI ^3^ at cholecystectomy	28.0 [26.0–31.0]	27.5 [26.0–31.0]	28.0 [26.0–35.0]
BMI ^3^ at bariatric surgery	NA	42.9 [38.5–44.8]	NA
Days between bariatric surgery and cholecystectomy	NA	492 [261–716]	NA

Values are medians and interquartile ranges. ^1^ BAR: bariatric gallstone patients; ^2^ Non-BAR: nonbariatric gallstone patients; ^3^ BMI: body mass index in kg/m^2^, NA: Not applicable.

## Data Availability

Because of the participant consent obtained as part of the recruitment process. The lipidomics data presented in this study are available on request from the corresponding author.

## Supplementary Materials

The following are available online at www.mdpi.com/article/10.3390/metabo11100682/s1, Figure S1: Partial least squares regression discriminant analysis, Figure S2: Heat-map of the most important (top 50) changed lipid species based on their *p*-value, Table S1: Identified lipid species.
