# Supplementary material for: Gallstone Formation Follows a Different Trajectory in Bariatric Patients Compared to Nonbariatric Patients
Source: Metabolites. 2021 Oct 5;11(10):682. doi: 10.3390/metabo11100682 (PMC8541369; doi:10.3390/metabo11100682)
Supplement: Supplementary file 1 [file metabolites-11-00682-s001.zip › Supplemental Figure S2.pdf]

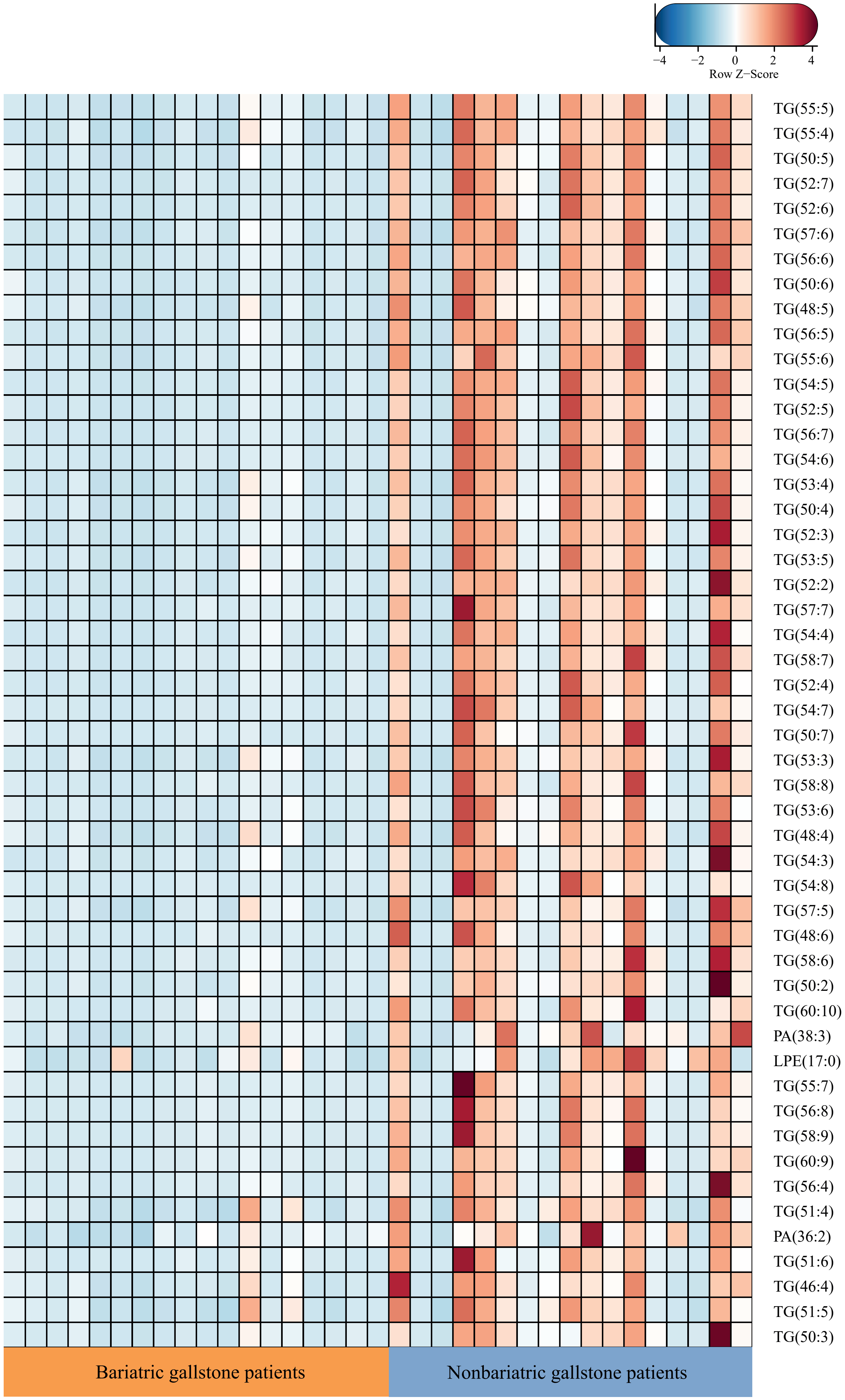

Figure S2. Heat-map of the most important (top 50) changed lipid species based on their p value. The heat-map was created by calculating the Z score of each individual sample in a row. The Z score indicates how many standard deviations each value deviates from the mean for that specie and is represented as color in the heat-map.
