# Supplementary figures and images for: Gallstone Formation Follows a Different Trajectory in Bariatric Patients Compared to Nonbariatric Patients
Source: Metabolites. 2021 Oct 5;11(10):682. doi: 10.3390/metabo11100682 (PMC8541369; doi:10.3390/metabo11100682)

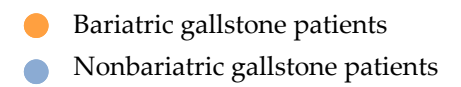

Supplement: Supplementary file 1 [file metabolites-11-00682-s001.zip › Supplemental Figure S1.pdf]
